# *Allium sativum* Compared to Cilostazol as an Inhibitor of Myointimal Hyperplasia

**DOI:** 10.5935/1678-9741.20160069

**Published:** 2016

**Authors:** Paulo Roberto da Silva Lima, Francisco Chavier Vieira Bandeira, Janio Cipriano Rolim, Manuel Ricardo Sena Nogueira, Mizael Armando Abrantes Pordeus, Andressa Feitosa Bezerra de Oliveira, Guilherme Benjamin Brandão Pitta

**Affiliations:** 1 Federal University of Alagoas (UFAL), Maceió, AL, Brazil and Centro de Angiologia e Cirurgia Vascular (Ceangio), João Pessoa, PB, Brazil.; 2 Department of Surgery of the Federal University of Paraíba (UFPB), João Pessoa, PB, Brazil.; 3 Vascular surgery in the Emergency Hospital and Trauma Dom Luiz Gonzaga Fernandes, Campina Grande, PB, Brazil.; 4 Federal University of Paraíba (UFPB), João Pessoa, PB, Brazil.; 5 Universidade Estadual de Ciências da Saúde de Alagoas (UNCISAL), Maceió, AL, Brazil.

**Keywords:** Hyperplasia, Garlic, Rabbits

## Abstract

**Objective:**

Intimal hyperplasia is associated with graft failure and vascular sutures in the first year after surgery and in postangioplasty restenosis. *Allium sativum* (common garlic) lowers cholesterol and has antioxidant effects; it also has antiplatelet and antitumor properties and, therefore, has great potential to reduce or inhibit intimal hyperplasia of the arteries. Our objective is to determine if the garlic has an efficacy to inhibit myointimal hyperplasia compared to cilostazol.

**Methods:**

Female New Zealand rabbits were divided into the following groups (n=10 each) according to treatment: group A, garlic, 800 µg×kg-^1^×day-^1^, orally; group C, cilostazol, 50 mg.day-^1^, orally; group PS, 10 ml of 0.9% physiological saline solution, orally. Our primary is the difference of the mean of myointimal hyperplasia. Statistical analysis was performed by using ANOVA and Tukey tests, as well as the Chi-square test. We calculated the 95% confidence interval for each point estimate, and the *P* value was set as < 0.05.

**Results:**

Group PS had a mean hyperplasia rate of 35.74% (95% CI, 31.76–39.71%); group C, 16.21% (95% CI, 13.36–19.05%); and group A, 21.12% (95% CI, 17.26–25.01%); *P*<0.0001.

**Conclusion:**

We conclude that *Allium sativum* had the same efficacy in inhibiting myointimal hyperplasia when compared to the positive control, cilostazol.

**Table:** 

Abbreviations, acronyms & symbols	
CI	= Confidence interval
Group A	= Group *Allium sativum* (Garlic)
Group C	= Group cilostazol
Group PS	= Group physiological saline
HDL	= High density lipoprotein
LDL	= Low density lipoprotein
RBP	= Rated burst pressure
UFPB	= Federal University of Paraíba
VLDL	= Very low-density lipoprotein

## INTRODUCTION

Intimal hyperplasia is the universal response of vessels to a chronic structural change that occurs in denuded arteries, arterialized veins, and in the anastomoses of prostheses used as grafts for bypass; it is also defined as an abnormal migration and proliferation of smooth vascular muscle cells associated with the deposition of extracellular connective tissue matrix, which is then accompanied by remodeling of the new tissue^[1]^.

Intimal hyperplasia has been recognized as a complication of arterial reconstructions since 1906^[2]^. The biology of intimal hyperplasia has many of the characteristic features of wounds, *e.g.*, the inflammation process^[3]^.

There is a great need for ways to prevent or reverse intimal hyperplasia. The myointimal hyperplasia is reported to be reversible only in the first and second weeks after vascular injury^[4]^, and it depends on the degree and duration of the causative injury^[2]^.

On the basis of the studies available in the literature^[5-9]^, we found that *Allium sativum* has beneficial effects such as anti-inflammatory, antioxidant, hypocholesterolemic, antitumoral, and anti-atherosclerotic actions, which prompted us to test its protective effect against myointimal hyperplasia. Our aim is to determine the mean difference of post-angioplasty myointimal hyperplasia in the external iliac artery of rabbits with induced atherosclerosis and treated with *A. sativum* compared with those treated with cilostazol, since the articles found in the literature^[10,11]^ demonstrate that cilostazol has effects of reducing the total cholesterol, triglyceride and phospholipids in serum, and moreover, the triglyceride content in the atherosclerotic arteries, so cilostazol significantly reduced the intimal atherosclerotic area.

## METHODS

This study was approved by the research ethics committee of the State University of Health Sciences of Alagoas (UNCISAL), Maceió, AL, Brazil, under number 63-A.

This is an experimental study^[12]^ in laboratory animals for 35 days, including 30 adult female New Zealand rabbits (*Oryctolagus cuniculus*; > 4 months old, >2 kg body weight). The animals were equally divided into 3 groups: PS group (physiological saline solution, negative control), group C (cilostazol, positive control), and group A (*A. sativum*, study drug).

The animals were underwent to experimental atherosclerosis and submitted to induction of myointimal hyperplasia in the right external iliac artery by angioplasty^[13-18]^.

The exclusion criteria were as follows:

- Older than 6 months;
- Animals that have acquired some disease in the quarantine period;
- Anatomical changes of the studied structures found during the angioplasty.

The selected rabbits comprised a nonprobability sample of convenience. The animals were grouped by using simple randomization with the help of a free research randomizer program (http://www.randomizer.org/form.htm), by using three blocks containing 10 numbers each. Each block corresponded to the drug used, and the numbers generated corresponded to the animals. Thus, each animal was assigned a letter and a number corresponding to the group and animal identification (*e.g.*, C1=animal 1, cilostazol group; A2=animal 2, *A. sativum* group). This identification was written with indelible blue ink on the inside face of the base of the right ear of the animals.

We opted for closed animal handling, and the trial was performed in the animal house of origin, under a forced exhaust ventilation system, with periods of natural luminescence, mean temperature of 20ºC, minimum noise, and humidity of around 50%. The animals were kept in appropriate cages with an area of 0.64 m^2^; they had no contact with their natural secretions. The diet consisted of water and granulated commercial diet (Purina^®^ for rabbits—15% crude protein, 2.5% ethereal extract, 16% fiber material, 10% ash, 2.5% calcium, 0.42% phosphorus, 13% humidity, 31.58% carbohydrates) given *ad libitum* before and during the experiment.

The rabbits were weighed weekly for the assessment of their nutritional status.

The following laws were followed in this study:

- Law 6638, May 8, 1979—Standards for Educational and Scientific Practice of Animal Vivisection;
- Universal Declaration of Animal Rights, UNESCO, October 15, 1978;
- Law of environmental crimes (Law No. 9,605/1998);
- The guidelines from Directive 2010/63/EU of the European Parliament on the protection of animals used for scientific purposes;
- The rules of the Brazilian College of Animal Experimentation—COBEA, 1991, on the Ethical Principles in Animal Experimentation.

All groups were offered 20 mL sifted yolk of chicken egg daily, administered orally in the morning with the aid of a 20 mL syringe, for a period of 100 days^[16]^. The diet was prepared daily, and proper hygiene was maintained to prevent contamination; the diet was given raw.

After 100 days of administration of the atherogenic diet, it was stopped and myointimal hyperplasia was induced by means of the following technique: rabbits were weighed and anesthetized with 10 mg.kg^-1^ xylazine and 40 mg.kg^-1^ ketamine, intramuscularly, on the proximal side to the right hindpaw, according to a technique described in the literature^[13,15,16,18]^.

Anesthesia was verified by the absence of pain reflex in the direct interdigital hold in the right ear. After anesthetic induction, trichotomy was performed in the lower abdomen and right inguinal region, followed by proper cleaning of the area, under aseptic conditions and antisepsis with polyvinyl pyrrolidone iodine degerming solution with 10% active iodine.

We collected 5 mL blood from the central ear vein with a syringe, to 6-mL tubes with a suitable preservative for biochemical analysis. After collection, the samples were sent for testing in the analysis laboratory of the Hospital University Lauro Wanderley at the Federal University of Paraíba (UFPB), where they were centrifuged and the low density lipoprotein (LDL), very low-density lipoprotein (VLDL), triglycerides, high density lipoprotein (HDL), and total cholesterol levels were analyzed. Subsequently, a 2–3-cm skin incision was made in the longitudinal direction in the inguinal fold with scalpel blade (#11) to expose the femoral artery, which was isolated with a 3-0 cotton thread. A small transverse arteriotomy was performed with a scalpel blade (#11) for the introduction of a 0.014-in metal guidewire and a semi-compliant catheter balloon (2.5 mm diameter and 20 mm long; balloon/artery ratio, 2.5:1), which was inserted in the lumen of the femoral right artery in the cranial direction and its position in the right external iliac artery was determined through visualization of the proximal balloon shoulder to the dissected inguinal ligament. The balloon catheter was inflated for 1 min until the rated burst pressure (RBP) of the angioplasty balloon (about 12 atm), leading to distension of the arterial wall. After balloon deflation and removal of all guidewire balloons, we performed ligation with a 3-0 cotton thread above and below the arteriotomy. Finally, the skin was sutured with a 4-0 nylon thread. Analgesics (10 mg.kg^-1^ ibuprofen orally, diluted in drinking water for 2 days) and antibiotics (20 mg.kg^-1^ ceftriaxone, intramuscular, single dose) were administered to all groups in the postoperative period, to prevent animal suffering. Twentyfour hours after surgery, the drug administration began in each group^[13-16]^.

Group A (*A. sativum*) received 800 µkg^-1^.day^-1^ of *A. sativum*^[5,7]^ by garage (the garlic was crushed and mixed with drinking water to a total volume of 10 mL) for 5 weeks, the PS group (negative control) received 0.9% physiological saline by gavage 10 mL.day^-1^ for 5 weeks and group C (cilostazol) received cilostazol (ZHENJIANG Haisen, China) at a dose of 50 mg.day^-1^^[19]^ for 5 weeks by gavage (cilostazol was diluted in 10 mL drinking water).

The drugs were administered during a 5-week period, ending when the animals were anesthetized by using the abovedescribed technique, and new blood samples were collected for post-dose drug lipid profile analysis. The animals were euthanized with a lethal dose of an anesthetic (150 mg.kg^-1^ pentobarbital^[19,20]^. The right external iliac artery, which received balloon injury due to the surgical procedure, was collected transabdominally.

Arterial blocks were fixed in 10% formalin for at least 24 h and then prepared for routine light microscopy, as follows: gradual and increasing dehydration with 70% alcohol until absolute alcohol concentration was reached; diaphonization in xylene and liquid paraffin embedding at 60°C; and preparation of paraffin blocks. The paraffin blocks were cut with a microtome in a thickness of 3 µm, and the sections were mounted on extrafine glass slides (76´ 25 mm). Next, they were stained with hematoxylineosin for general morphology study^[18]^; Verhoeff's stain for the evaluation of elastic fibers; and Masson's trichrome stain for general morphological study and characterization of elastic fibers, collagen, and muscle. Then, they were mounted with coverslips and natural resin. Also, immunohistochemistry assays were performed with the monoclonal HHF35 antibody^[15,18,21]^ (Kit DAKO Monoclonal Mouse anti-human Muscle Actin, clone HHF35) for determining the invasive reactions in the artery layers (smooth muscle), and C4d^[22,23]^ (Kit Spring Bioscience Rabbit Antihuman C4d Polyclonal antibody) to study the immune response in the vascular endothelium; in both immunohistochemistry tests, peroxidase was used as a developer. The slides were prepared and examined under magnifications of 2.5´, 10´, and 40´ by a duly accredited pathologist.

For morphometry, a Bioval^®^ optical microscope was used, with the program ToupTekView^®^ version ´86 3.7.2270 (copyright 2003–2013; http://www.touptek.com) and the Opticam^®^ 14 MP digital camera. The percentage of myointimal area was calculated by using the ImageJ 64 program, where the image of the vessel was formatted as follows:

- The area of the vessel adventitia was manually removed externally bypassing the muscle layer, which is easily visible. The automatic deletion was cutting structures of the muscle layer when some areas of adventitia had the same or similar color intensity that the middle layer (muscle);
- The remaining image was transformed into 8-bit;
- The rest of the image formed by the lumen of the vessel and myointimal layer was transformed into a mask, and the area in pixels was measured; this area was called the total area;
- Then, only the area of the vessel lumen was measured;
- The area of myointimal layer was calculated in pixels by subtracting the light area from the total area (area of the mean-intima layer with light);
- The percentage was then calculated by dividing the area of the myointimal layer by the total area.

For stereoscopy of the immunohistochemical results (monoclonal antibody HHF35 and C4d), we took three random field photos by using the 40X objective of microscope, and with each histological slide of each animal in each group. These photos do not suffer any further increase in its size. Then, the 36-point test^[24]^ was used, in which the area of immunomarkers was calculated by using the formula:

A = Vv/2Qa µm^2^
where Vv (volume density of the immunomarker) was estimated by counting the points (36-point test).
Vv = Pp/Pt
where Pp is the number of points that touch the immunomarkers, and Pt is the total number of points in the test area of 200 µm^2^, which in our case was 36 points.
Qa (number density of nuclei area) was calculated by the formula
Qa = N/At
where N is the number of nuclei counted in the test area and At is our test area, which was 200 µm^2^.

Masking for microscopy was performed by changing the letter and the initial marking number with another letter and number known only to the principal investigator. The new description was stored in an opaque and sealed envelope, which was only opened at the time of data analysis. The pathologists did not know which method was being used in the target vessel.

The sample size was arbitrated within thirty rabbits, 10 (ten) for each group studied; since in the literature there are studies that prove acceptable statistical results with fewer animals in the experiment with similar animal model^[5,15,17,20]^.

We also measured the sample size using the online calculator LEE (Epidemiology and Statistics Laboratory), available for free on the website: http://www.lee.dante.br/pesquisa/amostragem/di_1_pro_tes.html, which resulted in n = 9 (nine) for each group. To calculate the hypothesis test for a proportion the following parameters were used:

- Proportion of the population: 0.5% as suggested by the calculator.
- Proportion suggested in the survey: 30%, a value arbitrary.
- Significance level: 5%.
- Test power: 95%
- Hypothesis testing: two-tailed.

Data were collected in a standardized form and stored in a spreadsheet (Microsoft Excel^®^ 2011 for Mac, USA). Data entries were made independently.

Descriptive analysis was performed by calculating the 95% CI for each estimated point. Calculations were performed with the aid of statistical program Prism 6 for Mac OS X version 6.0b (October 3, 2012). Our hypotheses were as follows:

- H_O_: M_A_ = M_C_ = M_S_ (the frequency difference of the mean myointimal hyperplasia is the same among the studied drugs);
- H_1_: MA^1^ MC^1^ M_S_ (the frequency difference of the mean myointimal hyperplasia is different between groups.)

ANOVA tests were used (one-way and two-way)^[20]^ with confirmatory testing: Tukey (multicomparative) for quantitative data and the Chi-square test for qualitative data, both with two-tailed hypothesis. Where the two-way analysis of variance test was used for continuous temporal variables and the one-way test was used for transverse continuous variables. Furthermore, the Tukey test was used for quantitative data (continuous variables) because there was more than one group with different drugs^[20]^, whereas the χ^2^ test was used for qualitative data (nominal categorical variables).

We use a value of alpha (α) < 0.05 in the statistical test to reject the null hypothesis.

## RESULTS

**Fig. 1:**
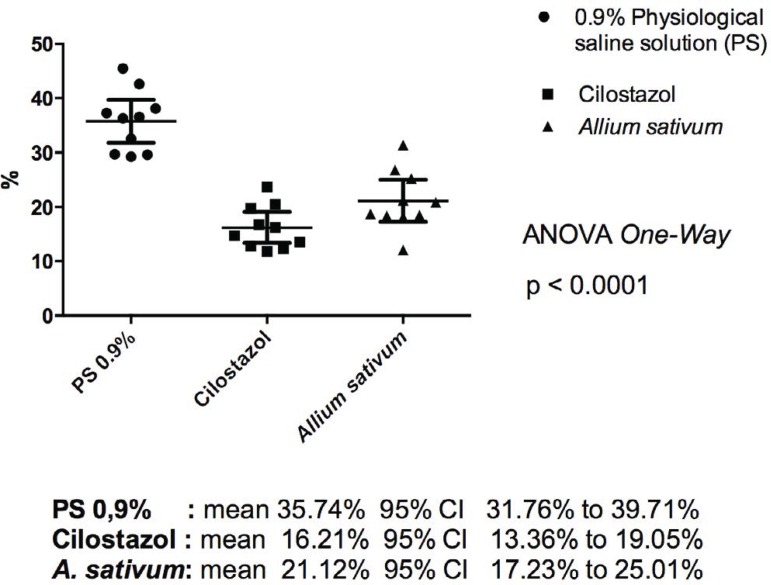
Mean rate of myointimal hyperplasia.

**Fig. 2:**
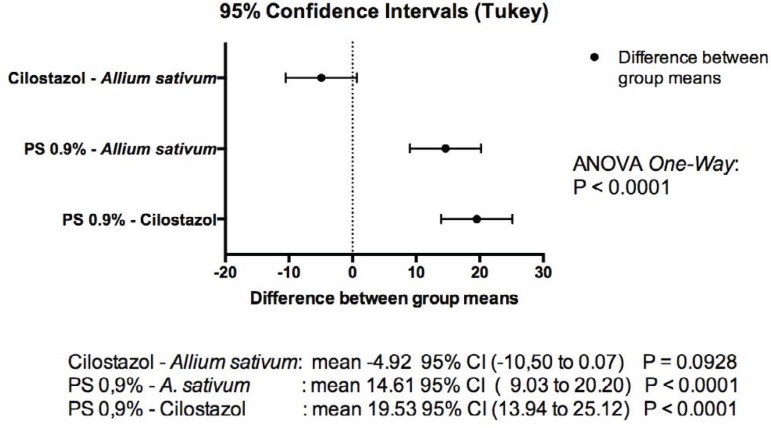
Difference between the means of groups (myointimal hyperplasia).

For the primary variable (the difference of the mean myointimal hyperplasia), the following results were obtained (Figures 1 and 2):

- Cilostazol x *A. sativum*: mean -4.92 95% CI (-10.50 to 0.07) *P*=0.0928;
- PS 0.9% x *A. sativum*: mean 14.61 95% CI (9.03 to 20.20) *P*<0.0001;
- PS 0.9% x Cilostazol: mean 19.53 95% (13.94 to 25.12) *P*<0.0001.

**Fig. 3:**
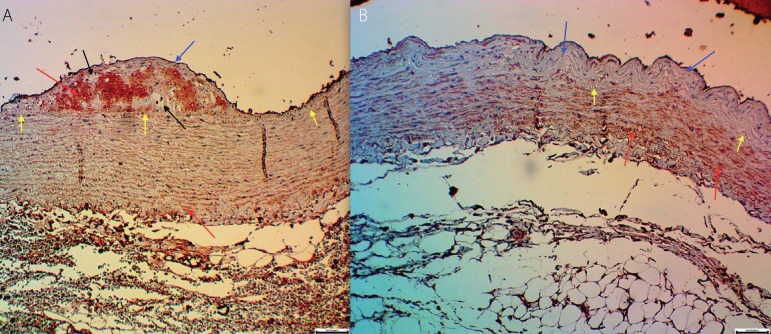
A: Image showing hyperplasia with invasion in the intima, yellow arrows show the internal elastic membrane, black arrows show foam cells and red arrows show positivity to HHF35 antibody in the intimal hyperplasia. Scale 5/100 mm, 10X. B: Image showing hyperplasia without muscle invasion in the intimal, blue arrows indicate hyperplasia without muscle invasion. HHF35 monoclonal, scale 5/100 mm, 10X).

Considering the area of intrusion or modification of myointimal cells in the muscle layer, characterized by the immunohistochemical marker HHF35 monoclonal antibody both hyperplasia with invasion in the intima (Figure 3A), and, in and hyperplasia without muscle invasion in the intima (Figure 3B) were observed. The following results were obtained:

- Negative control group, PS: mean, 8.35 µm^2^; 95% CI, 5.74–10.96 µm^2^;
- Positive control group, cilostazol: mean, 14.47 µm^2^; 95% CI, 8.06–20.88 µm^2^;
- Treated group, *A. sativum*: mean, 11.52 µm^2^; 95% CI, 7.09–15.96 µm^2^, with *P*=0.1387 and not significant in the Tukey test.

**Fig. 4:**
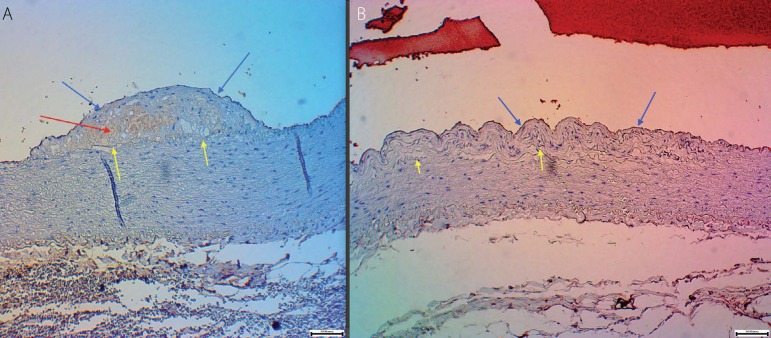
A: Image showing hyperplasia with active inflammation in the intimal indicated by red arrows – C4d positive, yellow arrows show the internal elastic membrane, red arrows show positivity to C4D polyclonal and blue arrows indicate hyperplasia, scale 5/100 mm, 10X. B: Image showing hyperplasia without active inflammation in the intima, yellow arrows show the internal elastic membrane and blue arrows indicate hyperplasia, scale 5/100 mm, 10X).

Considering the inflammatory area by complement and characterized in this study by the immunohistochemical marker C4d, hyperplasia with active inflammation in the intimal (Figure 4A) and hyperplasia without active inflammation in the intima (Figure 4B) were observed. The following results were obtained:

- Negative control group, PS: mean, 3.50 µm^2^; 95% CI, varying from 2.11 to 4.89 µm^2^;
- Positive control group, cilostazol: mean, 3.98 µm^2^; 95% CI, varying from 2.83 to 5.14 µm^2^;
- Treated group, *A. sativum*: mean, 4.16 µm^2^; 95% CI, varying from 1.66 to 6.67 µm^2^;
- with *P*=0.8295 and not significant in the Tukey test.

**Fig. 5:**
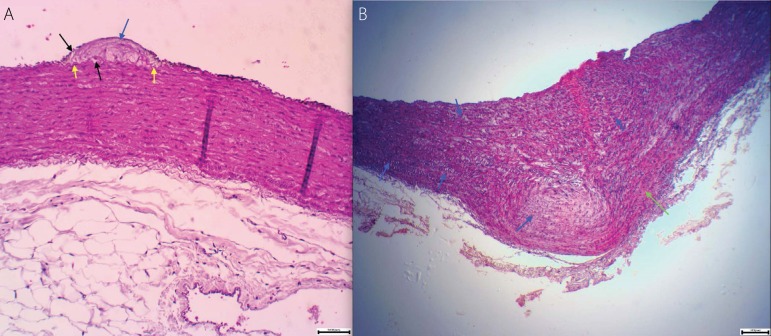
A: Isolated intimal hyperplasia. The blue arrows indicate hyperplasia, black arrows indicate the foam cells. Masson staining, 10X, scale 5/100 mm). B: Calcification in the middle layer. Blue arrows show the calcifications, green arrows indicate the normal muscle. Masson staining, 4X, scale 1/10 mm).

From the morphological results, we observed that there was no standardization of myointimal hyperplasia, as in some animals, hyperplasia occurred only in the intimate layer characterized by its elevation with invasion to the vessel lumen and the presence of foam cells without muscle invasion and other animals with muscle invasion (Figure 5A). Some animals showed changes only in the muscle layer (middle), and the arterial intimal layer was spared; these changes were characterized by calcium deposition at different levels (Figure 5B). Considering the immunohistochemical analysis with the HHF35 monoclonal antibody for the muscle cells, there was also no myointimal hyperplasia pattern, because we observed that the animals had hyperplasia with muscle invasion or muscle metaplasia in the intimate vascular network (Figure 3A), as well as no muscle change (Figure 3B).

By observing the immunohistochemical marker C4d (Figure 4), it was determined that there was no inflammatory standardization in the intima layer, considering that some animals had intimal hyperplasia without inflammatory reaction by the complement system and others with inflammation.

Concerning the mean difference of total cholesterol (Table 1) between the *A. sativum* and the 0.9% PS groups, we obtained 32.150 mg/dL with 95% CI varying from -41.384 to 105.684 mg/dL. The mean difference between the cilostazol and 0.9% PS groups was -26.750 mg/dL, with 95% CI varying from -100.284 to 46.784 mg/dL. However, between the cilostazol and *A. sativum* groups, the mean difference was -58.900 mg/dL with 95% CI from -132.434 to 14.634 mg/dL. From two-way ANOVA, we obtained *P*=0.0165; however, the results were not significant in the Tukey test. Thus, there was no difference between groups in total cholesterol.

**Table 1:** Absolute values of total cholesterol (mg/dL).

Animal	Cilostazol		*Allium sativum*		0.9% PS	
	Initial	Final	Initial	Final	Initial	Final
1	46	30	48	36	48	50
2	32	34	36	49	35	59
3	31	29	23	36	194	97
4	44	34	80	43	37	39
5	37	33	39	39	35	38
6	39	50	30	29	45	41
7	37	46	44	28	28	28
8	42	26	20	82	25	34
9	24	29	897	228	40	31
10	40	28	62	40	302	40
**Mean**	35.55 (95% CI 31.72 to 39.38)		94.45 (95% CI -23.41 to 212.3)		62.30 (95% CI 25.55 to 99.05)	

The difference of HDL cholesterol mean between *A. sativum* and 0.9% PS was 4.325 mg/dL with 95% CI varying from -6.667 to 15.317 mg/dL. The mean difference between the cilostazol and 0.9% PS groups was 0.570 mg/dL, with 95% CI of -10.422 to 11.562 mg/dL. On the other hand, between the cilostazol and *A. sativum* groups, the mean difference was -3.755 mg/dL with 95% CI of 7.237 to -14.747 mg/dL. From two-way ANOVA, we obtained *P*=0.0199; however, in the Tukey test, there was no significant difference between the groups.

For VLDL, the mean differences between the *A. sativum* and 0.9% PS, cilostazol and 0.9% PS, and cilostazol and *A. sativum* groups were infinitely in two-way ANOVA (*P*=0.8107); thus, there was no difference between groups concerning VLDL cholesterol. The Tukey test was not done, given that the ANOVA result was not significant.

Nevertheless, the mean difference in triglycerides between the *A. sativum* and 0.9% PS groups was 21.600 mg/dL, with 95% CI varying from -58.974 to 102.174 mg/dL. Between the cilostazol and 0.9% PS groups, the mean difference was -34.950 mg/dL, with 95% CI varying from -115.524 to 45.624 mg/dL. Between the cilostazol and *A. sativum* groups, the mean difference was -56.550 mg/dL with 95% CI of -137.124 to 24.024 mg/dL. The calculation with two-way ANOVA obtained *P*=0.2013, and the Tukey test was not significant; thus, there was no difference between groups.

Concerning the effect on the liver of the tested substances, *P*=0.5853 was obtained for the presence of steatosis in all groups, with a mean of five animals for the 0.9% PS group, with 95% CI of -58.53 to 68.53; a mean of five rabbits for the cilostazol group, with 95% CI varying from -45.82 to 55.82; and a mean of five rabbits for the *A. sativum* group, with 95% CI of -45.82% to 55.82.

Cholangitis of the liver occurred in a mean of five animals for the 0.9% PS group, with 95% CI of -7.70 to 17.71; a mean of five animals for the cilostazol group, with 95% CI of 5; and a mean of five rabbits for the *A. sativum* group, with 95% CI varying from -20.41 to 30.41 (*P*=0.6592).

Portal infiltration of the liver was found in a mean five animals for the 0.9% PS group, with 95% CI of 3.59 to 6.41; a mean of five animals for the cilostazol group, with 95% CI of 0.76 to 9.24; and a mean of five rabbits for the *A. sativum* group, with 95% CI varying from 2.18 to 7.82 (*P*=0.6211).

## DISCUSSION

We partially accepted hypothesis H1 because the results obtained in the experimental group (*A. sativum*) were similar to those obtained in the positive control (cilostazol), but differed from those obtained in the negative control (0.9% physiological saline solution). However, the tested drug had no effect on the inhibition of myointimal hyperplasia, since the result obtained was the same as the positive control.

*A. sativum* was chosen as the target drug in the study to inhibit myointimal hyperplasia because, according to Efendy et al.^[5]^, Campbell et al.^[7]^ and Yamaji et al.^[9]^, it has an anti-atherosclerotic effect. Moreover, according to Lau^[8]^, *A. sativum* has a hypocholesterolemic effect; furthermore, Borek^[6]^ reported that it has antitumor, anti-inflammatory, and antioxidant effects. Hence, there was a great possibility that *A. sativum* would act favorably in the inhibition of myointimal hyperplasia. The dose of 800 µg.kg^-1^.day^-1^ was chosen as the test dose in the present study, as Efendy et al.^[5]^ reported that 800 µg.kg^-1^.day^-1^ of crude extract of garlic can inhibit the development of fat layers and the accumulation of cholesterol in the vessel walls, thus protecting them against the development of atherosclerosis. The use of *A. sativum* in its original form as based on the study by Borek^[6]^, who found that allicin—a component of *A. sativum* — is unstable in solution, as it is a lipid-soluble, volatile organosulfur compound. Thus, it is essential that the maximum possible amount of the active substances of *A. sativum* is retained.

Rabbits were chosen for the study because, according to research performed by Ferrer et al.^[25]^, the rabbit is a valid atherogenic vascular injury model. Takagi et al.^[16]^ proved that the hyperplasia occurring in rabbits is similar to that occurring in human coronary arteries, besides Ylã-Herttuala et al.^[26]^ demonstrated that both rabbits and humans have a common pattern of inflammatory reactions in the complement system.

In particular, female rabbits were chosen because according to the literature, there is no difference in the use of female and male rabbits for this type of study^[20,25,27]^.

Egg yolk was chosen to induce atherosclerosis in female rabbits because Santos^[17]^ have demonstrated that this is a cheap and effective method.

The balloon diameter used was safe and effective, according to Ferrer et al.^[25]^, Gellman et al.^[13]^, and other researchers^[14]^.

The immunohistochemical study of specimens was performed with monoclonal HHF35^[15,18,21]^ and polyclonal C4d^[22,23]^ in an attempt to establish the mechanism of action of the drugs, given that the former is a marker for muscle cells and the C4d is a marker for the inflammatory process mediated by the complement system. Ylä-Herttuala et al.^[26]^ demonstrated the action of the complement system in atherosclerotic lesions and Tsai et al.^[19]^ identified the inhibitory activity of p38 by using cilostazol. Since the inhibition of p38 has been associated with possible therapeutic effects on autoimmune diseases and inflammatory processes^[28]^, we chose to assess the effect of *A. sativum* on inflammation through the complement system; then we used the marker C4d. However, in all animals, no statistical difference was observed with regard to muscle invasion in the intima as well as the inflammatory process mediated by the complement system in the myointimal wall. Therefore, the study was not able to characterize the manner in which the inhibition or decrease of myointimal hyperplasia occurred, given that there was no statistically significant inhibition of muscle invasion or reduction of the inflammatory process, with the use of *A. sativum* or cilostazol.

In the morphometric analysis, there was no pattern of hyperplasia among the groups because some animals had hyperplasia of the muscle layer with breakdown of muscle cells and calcium deposition; others only had a change of intima such as stratification of the intimal layer and muscle invasion; and others showed change in two layers, suggesting that there are various mechanisms underlying the development of myointimal hyperplasia, even though there were only two induction mechanisms — the hypercholesterolemic diet and endothelial injury (balloon). However, these findings are in contrast with those from the literature, which states that the distribution of hyperplasia may be intimal, diffuse, focal, or within the body vessel. Furthermore, Van Craeyveld et al.^[27]^ found that the main changes of atherosclerosis and hyperplasia actually occur in the deep layers of the vessel (muscle) and not in the intima. The staining method of Verhoeff, which is used for elastic fibers, was performed to try to define the intima and the muscle of vessel to calculate only the intimal hyperplasia; however, it was not effective in this delimitation. Therefore, we studied the myointimal layer.

*A. sativum* and cilostazol were able to inhibit myointimal hyperplasia in 40.9% and 54.64% of animals, respectively, as compared to the negative control (0.9% physiological saline solution), demonstrating the effectiveness of both substances. Although the proportion of animals exhibiting myointimal hyperplasia inhibition was 13.74% higher with cilostazol than with *A. sativum*, the fact that cilostazol is a drug that is already the active ingredient should be considered. Thus, even though a plant in its original form was used, without isolation of the active principle, a statistically similar result was obtained.

*A. sativum* and cilostazol were not able to reduce the lipid levels, thus our results are consistent with the current findings^[5,7,29]^ and we did not observe a difference in the lipid levels following in the dose of 800 µg.kg^-1^.day^-1^ in rabbits. Others studies^[29,30]^ indicated that the hypolipidemic action of garlic was observed only after 2 to 6 months of continuous administration of *A. sativum*, thus suggesting that the time used to conduct this study (35 days) was not sufficient to observe such a hypolipidemic effect, which was also reported by Ried et al.^[31]^.

With regard to the safety of *A. sativum*, the use of this drug in animals for the inhibition of myointimal hyperplasia was successful, without any difference in the presence of steatosis, cholangitis, and portal infiltration between groups. *A. sativum* has a LD50 of 3034 mg.kg^-1^ and maximum dosage without side effects of 2200 mg.kg^-1^^[32]^; moreover, it is not contraindicated in pregnant animals^[29]^.

The research carried out in this study paves the way for a new drug against myointimal hyperplasia, which is the main cause of failure of both coronary as well as peripheral angioplasties that lead to reinfarctions and failure of vascular grafts, and consequently to the need for amputations in many people, particularly diabetic patients.

This research forms the basis for further research, and allows for:

- Another study to be performed using higher doses of *A. sativum*, as this study used only the plant and not even an extract, and still matched the protective effect of an expensive drug, which can be useful in the third world population.
- Further research to be conducted with crude extract, followed by fractionation of this extract to obtain its active ingredient.

## CONCLUSION

*Allium sativum* had the same efficacy in inhibiting myointimal hyperplasia when compared to the positive control, cilostazol.

**Table:** 

Authors’ roles & responsibilities	
PRSL	Conception and design study; realization of operations and/ or trials; analysis and/or data interpretation; statistical analysis; final manuscript approval
FCVB	Realization of operations and/or trials; final manuscript approval
JCR	Realization of operations and/or trials; final manuscript approval
MRSN	Realization of operations and/or trials; final manuscript approval
MAAP	Realization of operations and/or trials; final manuscript approval
AFBO	Final manuscript approval
GBBP	Manuscript writting or critical review of its content; final manuscript approval
